# Understanding the Dynamic of POMS Infection and the Role of Microbiota Composition in the Survival of Pacific Oysters, Crassostrea gigas

**DOI:** 10.1128/spectrum.01959-22

**Published:** 2022-10-31

**Authors:** Lizenn Delisle, Olivier Laroche, Zoë Hilton, Jean-François Burguin, Anne Rolton, Jolene Berry, Xavier Pochon, Pierre Boudry, Julien Vignier

**Affiliations:** a Cawthron Institute, Nelson, New Zealand; b Institute of Marine Science, University of Auckland, Warkworth, New Zealand; c Département Ressources Biologiques et Environnement, Ifremer, ZI de la pointe du diable, Plouzané, France; Barnard College, Columbia University

**Keywords:** OsHV-1, Pacific oyster, POMS, microbiome, 16S rRNA gene sequencing, droplet digital PCR

## Abstract

For over a decade, Pacific oyster mortality syndrome (POMS), a polymicrobial disease, induced recurring episodes of massive mortality affecting Crassostrea gigas oysters worldwide. Recent studies evidenced a combined infection of the ostreid herpesvirus (OsHV-1 μVar) and opportunistic bacteria in affected oysters. However, the role of the oyster microbiota in POMS is not fully understood. While some bacteria can protect hosts from infection, even minor changes to the microbial communities may also facilitate infection and worsen disease severity. Using a laboratory-based experimental infection model, we challenged juveniles from 10 biparental oyster families with previously established contrasted genetically based ability to survive POMS in the field. Combining molecular analyses and 16S rRNA gene sequencing with histopathological observations, we described the temporal kinetics of POMS and characterized the changes in microbiota during infection. By associating the microbiota composition with oyster mortality rate, viral load, and viral gene expression, we were able to identify both potentially harmful and beneficial bacterial amplicon sequence variants (ASVs). We also observed a delay in viral infection resulting in a later onset of mortality in oysters compared to previous observations and a lack of evidence of fatal dysbiosis in infected oysters. Overall, these results provide new insights into how the oyster microbiome may influence POMS disease outcomes and open new perspectives on the use of microbiome composition as a complementary screening tool to determine shellfish health and potentially predict oyster vulnerability to POMS.

**IMPORTANCE** For more than a decade, Pacific oyster mortality syndrome (POMS) has severely impacted the Crassostrea gigas aquaculture industry, at times killing up to 100% of young farmed Pacific oysters, a key commercial species that is cultivated globally. These disease outbreaks have caused major financial losses for the oyster aquaculture industry. Selective breeding has improved disease resistance in oysters, but some levels of mortality persist, and additional knowledge of the disease progression and pathogenicity is needed to develop complementary mitigation strategies. In this holistic study, we identified some potentially harmful and beneficial bacteria that can influence the outcome of the disease. These results will contribute to advance disease management and aquaculture practices by improving our understanding of the mechanisms behind genetic resistance to POMS and assisting in predicting oyster vulnerability to POMS.

## INTRODUCTION

Since 2008, Pacific oyster mortality syndrome (POMS) has been a major challenge for the growth of Pacific oyster aquaculture in most production countries. Recurrent mass mortality events associated with ostreid herpesvirus 1 (OsHV-1) μVar have been recorded in numerous countries producing Crassostrea gigas, inducing 40% to 100% mortality in oysters less than 1 year old (1–3) but also affecting adult oysters in some cases (4–6). The first major outbreak was recorded in France during the summer of 2008 (7). The same year, OsHV-1 μVar was detected along the European coastline from southern Norway to Portugal (3, 8), and closely related variants were subsequently detected during massive mortality events in Australia (9), New Zealand (10), Korea (11), and more recently, California (12).

POMS is a polymicrobial disease caused by the combined development of viral and bacterial infections (13). Disease occurrence is multifactorial and depends on multiple factors influencing the host, the pathogens, and the environment. Among those, biological factors such as genetic background and developmental stage (14–17), metabolism and diet (18), and environmental factors, mainly the water temperature (6, 19–21), are the most prominent. Experimental challenges using oyster families with contrasted resistance to OsHV-1 recently led to a better understanding of the POMS infection process. Twelve hours after exposing the vulnerable oysters to OsHV-1 μVar, an intense viral replication was detected. At 48 h, the viral load and the transcriptional activity were maximal and remained stable until the first death at 66 h postinfection in vulnerable oysters. The viral gene expression induced an immunocompromised state that concomitantly allowed for a massive colonization of the gills by opportunistic bacteria evolving toward a bacteremia and leading to oyster death (13). In contrast, resistant oysters (i.e., which commonly experience very low mortality, less than 5%) weakly replicate OsHV-1 and do not show changes in their microbiota composition after being exposed to a high viral load (13, 17).

The role of the oyster microbiota in POMS is complex and ambivalent. For instance, microbiota composition is highly variable in relation to geographic location, seasonality, oyster age, tissue type, and health status (22–26). Changes in at least one of these parameters associated with an immunocompromised state in oysters can influence disease occurrence by inducing the replacement of benign microbial colonizers with a consortium of different pathogens (25). Administering antibiotic treatment early after OsHV-1 infection was shown to reduce mortality compared to untreated oysters, underlying the key role of bacteria in the pathogenicity of the disease (13). It has been demonstrated that an increase in bacterial load, mainly in the *Vibrio* community is concomitant with mortality during POMS (13, 26–28). Moreover, recent studies showed significant differences in the structure of the microbiome of oysters exhibiting various levels of susceptibility to POMS (22, 29). For example, the genera *Arcobacter*, *Marinomonas*, *Psychrobium*, *Psychromonas*, and *Vibrio* were identified as direct contributors to bacteremia after viral burst (13, 22, 27, 29). Some evidence also suggest that the microbiota might protect the host from pathogens (30), acting as a physical barrier by producing antimicrobial peptides (31, 32) or stimulating the immunity of their host (33, 34).

In New Zealand, the presence of a herpes-like virus was first reported in 1992 after massive mortality of 7-day old *C. gigas* larvae occurred in a hatchery (35). However, it was not until April 2010 that the first massive mortalities affecting juvenile oysters were observed, where multiple oyster translocations led to a fast spread nationwide. The OsHV-1 variant (GenBank accession number JN639858) identified in oysters during the 2010 outbreak presented typical μvar deletions in the C2-C6 region (open reading frame 4 [ORF 4]) but also shared two identical nucleotides with the reference strain (GenBank accession number AY509253) that differed from the variant μvar (GenBank accession number HQ842610) (10).

Little is known about the POMS infection process in New Zealand. A field study carried out in the summer of 2010 to 2011 recorded 14% spat mortality after 6 days of deployment on aquaculture farms, increasing to 50% after 9 days and 70% after 13 days—a relatively late dynamic of infection—with an increase in viral load and *Vibrio* detected in oysters after 4 days (10). These field-based observations suggest that further knowledge of the dynamics of POMS infection is necessary to improve POMS risk management but also to support the New Zealand oyster aquaculture industry and to sustain the development of new mitigation strategies in New Zealand.

In the present study, we primarily aimed to (i) describe the POMS infection process in juvenile New Zealand oysters and (ii) characterize the temporal dynamics of the microbiota associated with oysters after exposure to OsHV-1. We used a lab-based experimental approach of infection via immersion in water derived from infected donors, which reproduces the natural route of infection. We challenged 10 biparental oyster families displaying contrasted genetically based susceptibility to the POMS and combined various molecular analyses (viral load quantification, viral gene expression, and 16S rRNA gene sequencing) to describe the time course of infection and changes in microbial composition and histopathological assessments to describe the tissue response associated with POMS.

## RESULTS

### Survival and viral progression.

Survival of pathogen donors (oysters injected with OsHV-1 suspension) was significantly reduced at 48 h postinjection and reached 29% at 72 hours postinfection (hpi). By then, pathogen donors shed 2.64 × 10^9^ OsHV-1 DNA copies/L to the surrounding seawater (see Fig. S1 in the supplemental material).

**FIG 1:**
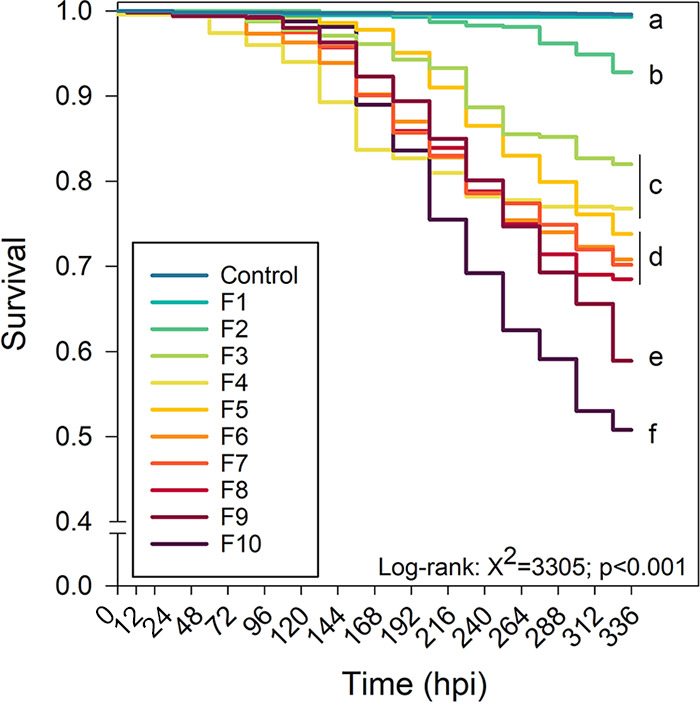
Survival of oysters (OsHV-1-exposed recipient oysters) in each family (F1 to F10) challenged with OsHV-1. “Control” group represents a mean survival of all recipient oysters challenged with SSW. Values are means (*n* = 3 replicate tanks) and letters indicate significant differences. Log-rank, Chi square = 3,305, *P* value < 0.001.

Survival of control recipient oysters was 100% irrespective of the family (Fig. 1). Very low levels of virus were observed in the control water samples (<1 × 10^2^ OsHV-1 DNA copies/L), likely the result of aerosolization of viral DNA during inoculation of OsHV-1 or during experimental handling. Because no virus was amplified from tissue of control recipient oysters and no control recipient died, only the pathogen-exposed recipient oysters will be considered hereafter.

Mortality started 48 hpi. Specifically, significant mortalities (>10%) were recorded 144 hpi in family 4 (F4), 168 hpi (F10), 192 hpi (F6, F7, F8, and F9), and 240 hpi (F5 and F3), whereas very low mortality (<10%) was observed for F1 and F2 (Fig. 1). At the end of the experiment (336 hpi), the final survival of pathogen-exposed recipient oysters ranked as follows: F1 (99.3% ± 0.4), F2 (92.8% ± 1.1), F3 (82.0% ± 2.3), F4 (76.8% ± 2.1), F5 (73.8% ± 2.2), F6 (71% ± 2.2), F7 (70.2% ± 2.3), F8 (68.5% ± 2.3), F9 (58.9% ± 2.4), and F10 (50.8% ± 2.5) (Fig. 1).

Based on this final survival, F1 and F2 were subsequently grouped and classified as “highly resistant”; F3, F4, F5, F6, F7, and F8 as “resistant”; and F9 and F10 as “vulnerable.” this classification (family type) will be used throughout.

At the onset of the experiment (i.e., prior to the transfer of infected waters), OsHV-1 DNA was not detected in the water from the recipient tanks (Table 1). Following infection, viral load in the water increased to reach 4.75 × 10^9^ (± 0.69 × 10^9^) copies · L^−1^ at 168 hpi. By 240 hpi, viral load decreased to 2.05 × 10^7^ (± 1.44 × 10^7^) copies · L^−1^ of water (Table 1). No significant differences were found in viral load between tanks over time (*P* = 0.709).

**TABLE 1:** Quantification of OsHV-1 DNA in water of recipient oysters^*a*^

Time (hpi)	OsHV-1 in water (copies · L^−1^)	Statistical difference^*b*^
0	0.00 ± 0.0	C
24	(3.87 ± 1.2) × 10^9^	A
48	(2.92 ± 1.68) × 10^8^	A
72	(6.65 ± 2.08) × 10^8^	A
96	(7.96 ± 3.34) × 10^8^	A
120	(5.41 ± 1.11) × 10^8^	A
168	(4.75 ± 0.69) × 10^9^	A
240	(2.05 ± 1.44) × 10^7^	B

a Quantity of viral DNA measured in the water of infected recipient tanks (*n* = 3). Data are expressed as copies of OsHV-1 per liter. Viral load was estimated before infection (0 hpi) and throughout.

b Different letters indicate statistical difference (*P* ≤ 0.05) between time points.

**FIG 2:**
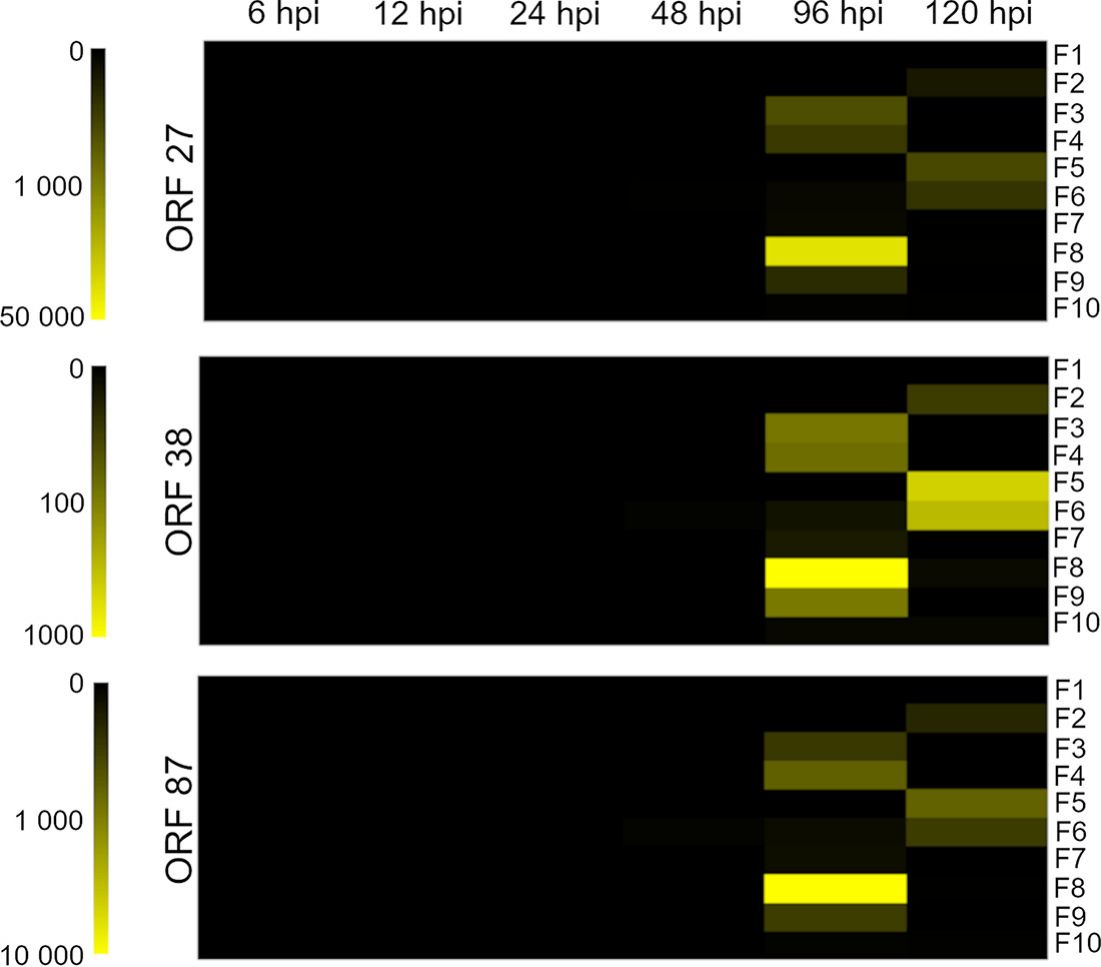
Heatmap presenting the relative expression of three OSHV-1 open reading frames—ORF 27, 38 and 87—expressed in oysters from 10 different families (F1 to F10) as a function of time in hours postinfection (hpi). Color intensity indicates the magnitude of ORF expression in copies per milligram of oyster.

Expression of the three OsHV-1 viral open reading frames (ORFs) varied significantly as a function of the ORF type, family, and time. Specifically, ORF 38 was expressed in all families from 96 hpi with low expression levels in F1 and F10 (Fig. 2). ORFs 27 and 87 were also expressed in most families, except for families F2 and F5 where viral gene expression started at 120 hpi and for F1 where ORF expression was never recorded (Fig. 2). Family 8 exhibited the highest viral transcription activity (Fig. 2), whereas ORF expression associated with F1, F7, and F10 remained the lowest over time, suggesting very little viral gene expression occurred in these family lines.

### Histological analyses.

**FIG 3:**
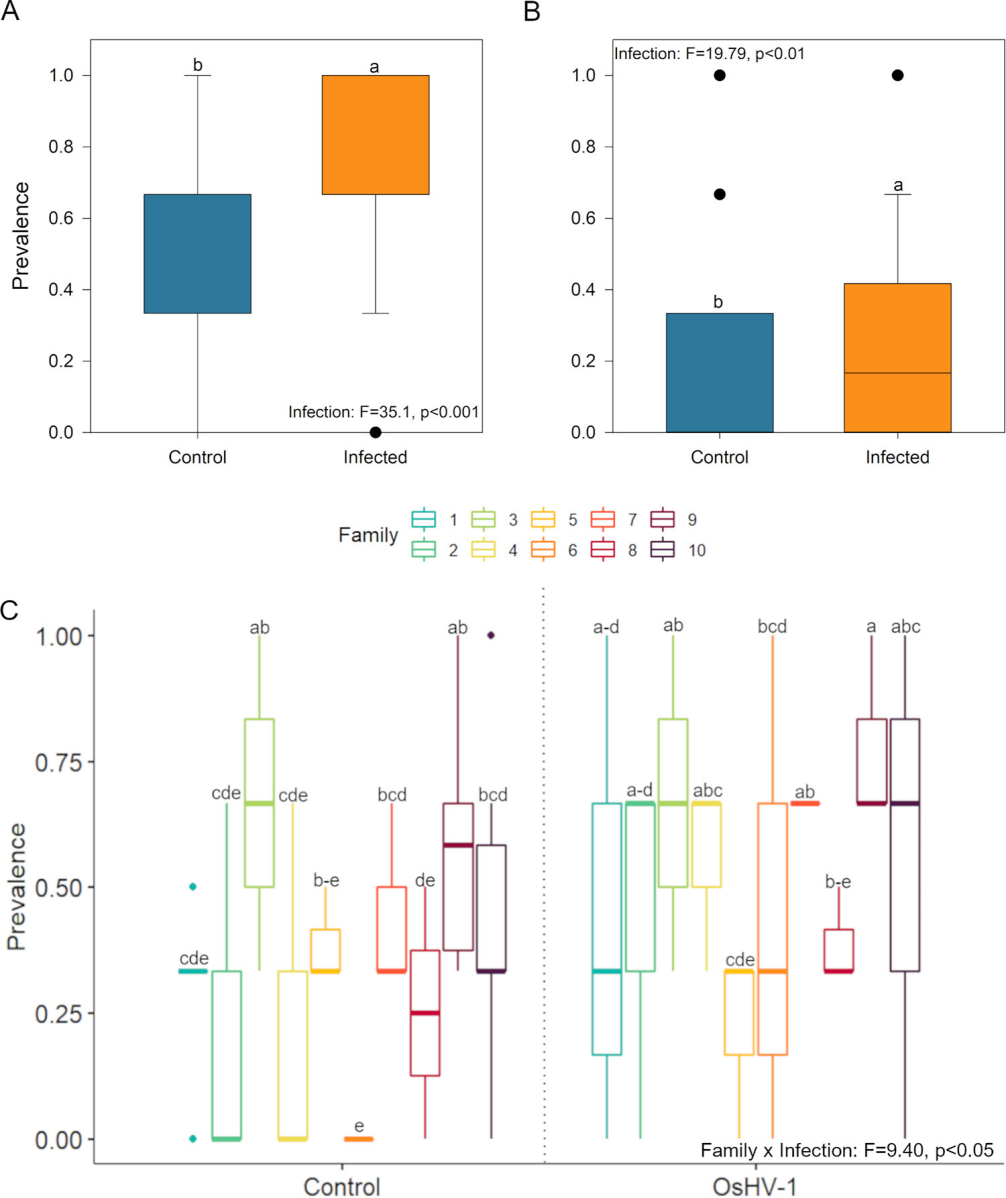
Histological assessments. The prevalence of “loose” connective tissue (A) and “blebby” hemocytes (B) in the digestive gland of control and OsHV1-infected oysters at 0 and 72 hpi (all families combined). (C) The prevalence of loose connective tissue in the mantle of oysters from all families (F1 to F10) assessed at 0 and 72 hpi in the control and OsHV1-exposed oysters. Treatments with the same letter were not significantly different (*P* > 0.05).

**FIG 4:**
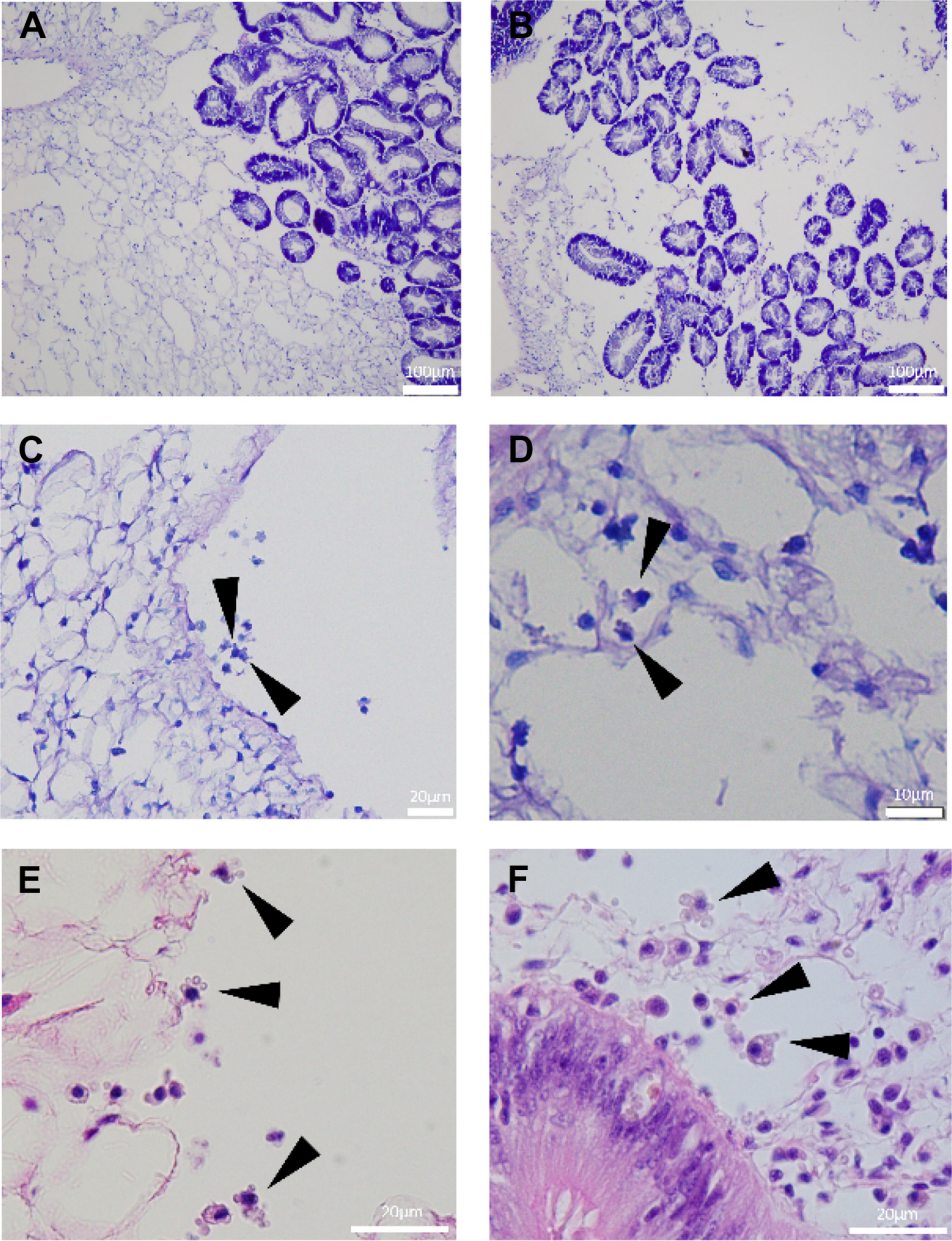
Examples of histopathological features observed in *Crassostrea gigas* spat infected with OsHV-1. An example of normal (A) and “loose” (B) connective tissue in the digestive gland, showing a loss of structure and large edematous areas around the digestive tubules. Examples of “blebby” hemocytes (black arrows) in a blood space in the digestive gland (C and E) and in the connective tissue around the digestive gland (D and F). Tissues in micrographs C and D are stained with Giemsa and those in E and F are stained with hematoxylin and eosin.

Significant differences (*P* < 0.05) in the prevalence of pathological features were observed in the mantle and digestive gland between oyster spat exposed and not exposed to OsHV-1 for 72 h (Fig. 3 and 4). Oysters exposed to OsHV-1 showed higher instances of “loose” connective tissue (CT) in the mantle (*P* = 0.0176, nested analysis of variance [ANOVA]) and digestive gland (*P* = 0.000147) (Fig. 3A) compared with controls. This loose CT tissue showed a loss of structure and large edematous areas (Fig. 4B) compared to normal CT (Fig. 4A). Significant differences in the occurrence of loose CT in the mantle were also observed between families (*P* = 0.0122), and there were interactive effects of infection and family (*P* = 0.0230) (Fig. 3C).

The presence of “blebby” hemocytes was significantly higher in the digestive gland of spat exposed to OsHV-1 (*P* = 0.00671) (Fig. 3B and Fig. 4C to F) compared with that in controls. These hemocytes showed multiple protrusions, which occasionally budded off from the cytoplasm. Giemsa staining revealed these protrusions to be mostly basophilic in nature (Fig. 4C); however, some were filled with acidic contents. For greater definition, further investigations with hematoxylin and eosin staining showed predominantly basophilic protrusions, some containing clear globules (Fig. 4E and F).

### Microbial analyses.

A total of 15,920,350 16S (V3-V4) rRNA reads (mean of 88,446 per sample) were sequenced, of which 78% remained after quality filtering, 70% after denoising and merging, and 66% after chimera removal (see Table S1 in the supplemental material). After discarding negative controls, removal of potential contamination, unidentified amplicon sequence variants (ASVs), nonbacterial ASVs, and rare ASVs further reduced read count by 0.15, 0.05, 0.08, 5.87, and 3.37%, respectively, for a total of 7,935,417 sequence reads (mean of 66,684 reads per sample) and a total of 3,011 ASVs.

### (i) Composition of bacterial communities and alpha diversity in oysters.

**FIG 5:**
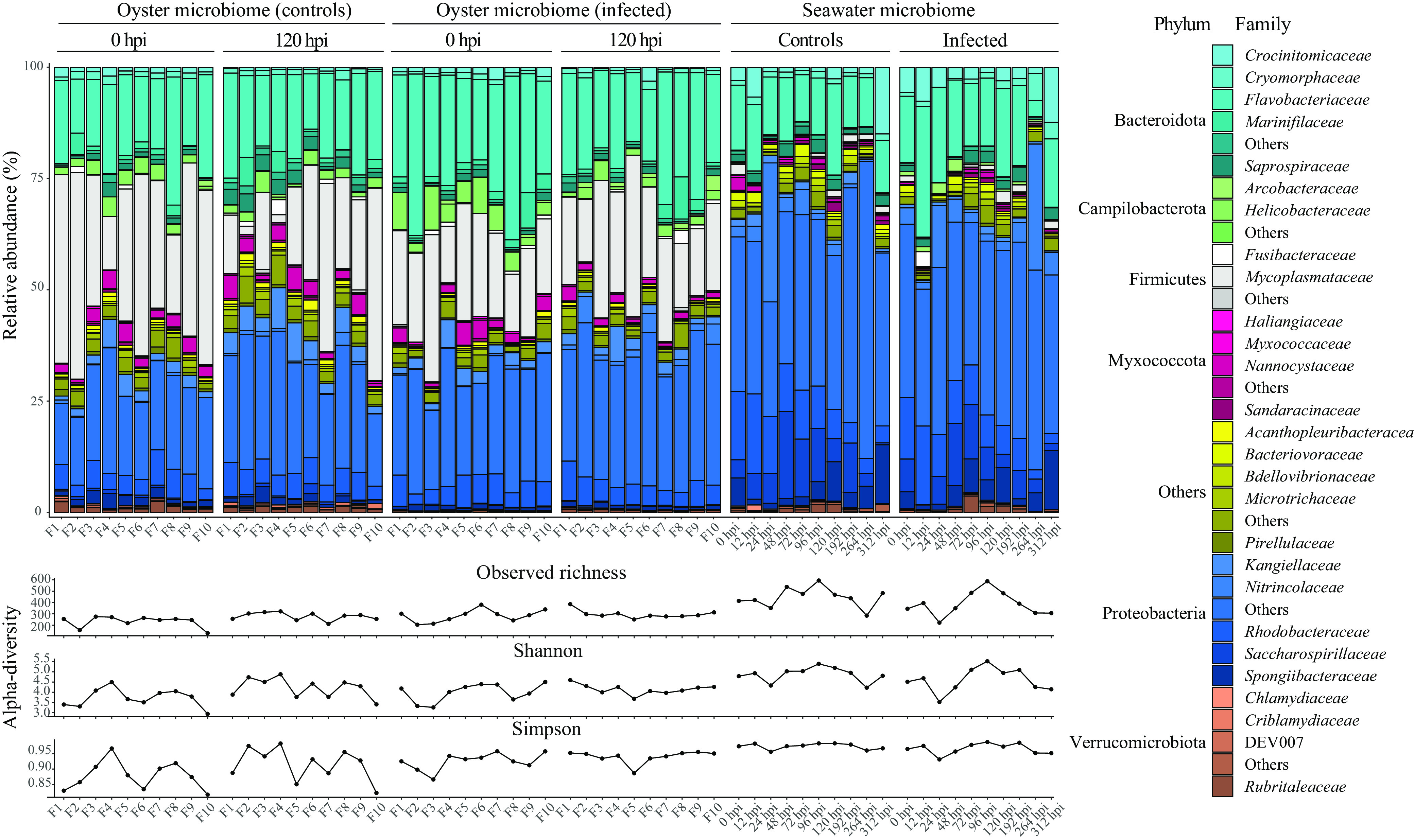
Relative abundance of the most important bacterial families and phyla (top) and alpha diversity (richness, Shannon, Simpson) (bottom) values per sample type (oyster microbiota versus seawater), treatment (control versus infected), and time. Bacterial families that were not among the first 5 most abundant families within each phylum were grouped under “Others.” Similarly, phyla that were not among the first 6 most abundant phyla were grouped under “Others.”

We first analyzed the microbiota composition in whole tissue from recipient oysters over the course of the experimental infection. When examined as relative abundance in whole tissue, oysters carried three main bacterial phyla, *Proteobacteria* (38%), *Bacteroidota* (27%), and *Firmicutes* (21%), with small fractions (<20%) of *Myxococcota*, *Campilobacterota*, and *Verrucomicrobiota*. The taxonomic profiles at family and phylum levels were noticeably different between oyster and seawater microbiota, with no discernible difference based on time or infection (Fig. 5).

Linear mixed-effects regression found no significant difference in alpha diversity between families and infection status. However, a significant increase in alpha diversity was noticeable over time for resistant and highly resistant families (control and infected) for both richness and Shannon index (see Table S2 in the supplemental material).

### (ii) Composition of bacterial communities and alpha diversity in water.

Relative abundance of the bacterial phyla in the surrounding water showed that *Proteobacteria* were the dominant phylum (>70%) followed by *Bacteroidota* (>20%, Fig. 5). Very low abundances of *Firmicutes* and *Campilobacterota* were detected in the water, indicating a specificity to oyster tissue.

Using linear mixed-effects regression, no significant effect of “infection” or “time” after start of experiment could be observed on the bacterial richness and Simpson index. Shannon index, however, did show a weak but significant negative effect (*P* value = 0.05; *R*^2^ = 0.06) of infection (see Table S3 in the supplemental material).

### (iii) OsHV-1 infection induces deep change in beta diversity.

**FIG 6:**
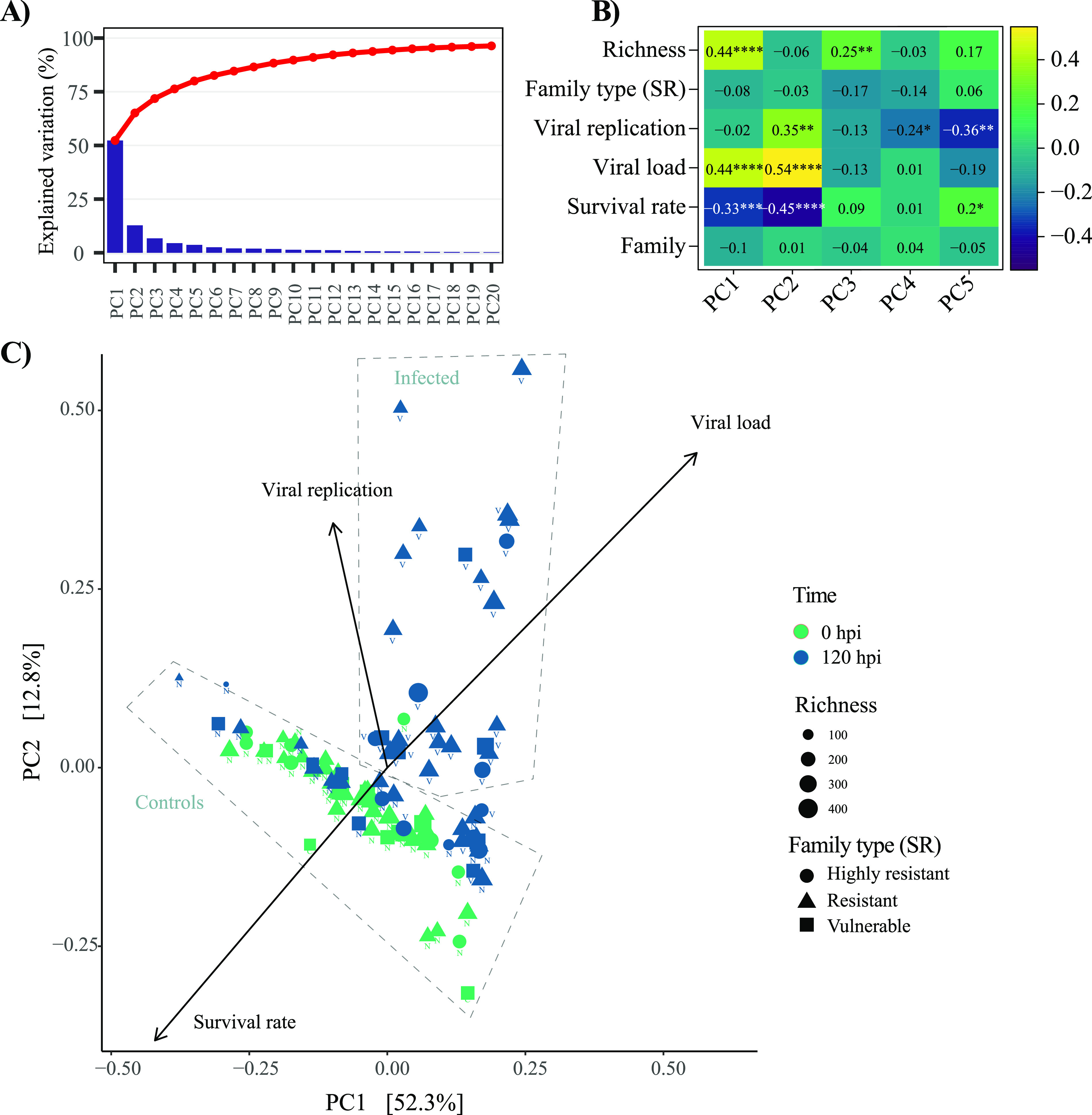
Beta diversity analysis of host microbiome using a principal-component analysis. (A) Percent of variance explained per principal component (PC). (B) Pearson correlation of variables of interest with each component with level of significance (*P* value) indicated by the number of asterisks (*, *P* = 0.05; **, *P* = 0.01; ***, *P* = 0.001; ****, *P* = 0.0001). (C) Principal-component analysis including overlaid variables such as viral load, viral gene expression, and survival rate associated with the first two components. N, noninfected oyster samples (control); V, virus-infected oyster samples (recipient oysters).

Beta diversity (herein the extent of change in community composition) was assessed with a principal-component analysis (PCA) to explore the potential influence of several factors, including bacterial richness, family type (SR), viral gene expression (ORF), viral load, and family. The PCA showed a clear clustering of host microbiota based on treatment (infected versus controls) with evidence of effect of viral load and viral gene expression on oyster microbial diversity and oyster survival rate (Fig. 6).

Permutational analysis of variances confirmed that infection (*P* = 0.001) and time (*P* = 0.001) played a significant role in structuring microbial community composition in oysters with no effect of family type or their interaction with infection (Table 2). Conversely, a significant difference in community composition could be observed between individual oyster families, but there was no significant interaction with these and infection (see Table S4 in the supplemental material).

**TABLE 2:** Permutational analysis of variance of oyster microbial community composition with infection, family type, time, and their interaction terms as factors

Term	*R* ^2^	*P* value^*a*^
Infection	0.097	**0.001**
Family type	0.019	0.306
Time	0.037	**0.001**
Family type × infection	0.017	0.371
Collection date × family type	0.013	0.852
Residuals	0.461	

a Significant *P* values are highlighted in bold.

### (iv) Core microbiome analysis.

Analysis of the core microbiota indicated that, overall, 14 bacterial genera were only carried by highly resistant oysters and 20 by vulnerable oysters (see Fig. S2 and Table S5 in the supplemental material). Specifically, core analyses revealed that the microbiome of oysters was dominated by *Polaribacter* and *Aquimarina*, with marginal differences between vulnerable and highly resistant oyster families (see Fig. S3A in the supplemental material). Among these differences, *Acanthopleuribacter*, *Cohaesibacter*, *Marinifilum*, *Mycoplasma*, *Roseovarius*, *Vibrio*, and *Vicingus* were more prevalent in highly resistant families, while *Aquibacter*, *Amphritea*, BD1-7, *Flavirhabdus*, *Fusibacter*, *Salinirepens*, Woeseia, and SVA0996 were more prevalent in vulnerable families (Fig. S3B).

### (v) Identification of bacteria associated with the disease and their potential use to predict oyster mortality.

**FIG 7:**
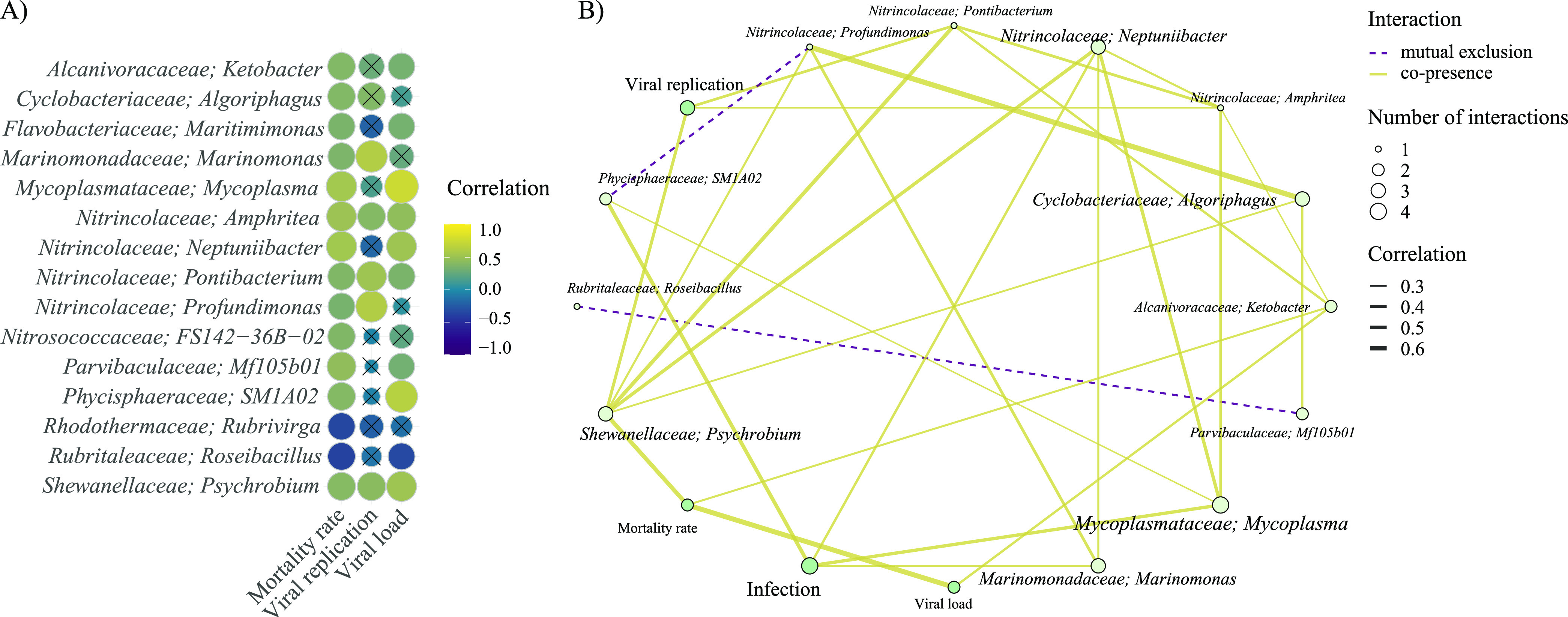
(A) Pearson correlations of centered-log transformed genera abundance with mortality rate, ORF, and viral load; (B) network associations between these bacteria and with mortality rate, viral gene expression, viral load, and treatment. Only correlations with an absolute value of >0.25 with mortality rate are shown in panel A, and only those above 0.25 are shown in panel B. The size of variables (points and text) in panel B is proportional to the number of significant associations (*r* > 0.25) that they have with other variables.

Pearson correlations of the centered-log ratio abundance of bacterial genera showed a positive association of *Ketobacter*, *Algoriphagus*, *Maritimimonas*, *Marinomonas*, *Mycoplasma*, *Amphritea*, *Neptuniibacter*, *Pontibacterium*, *Profundimonas*, Mf105b01, SM1A02, and *Psychrobium* with mortality rate and viral gene expression and/or viral load (Fig. 7). Conversely, the presence of *Rubrivirga* and *Roseibacillus* was negatively correlated with mortality rate and/or viral load (Fig. 7A). Network analysis showed significant positive interactions between several of these bacteria, including *Psychrobium* with *Profundimonas*, *Neptuniibacter*, and *Algoriphagus* and between *Mycoplasma*, *Neptuniibacter*, and *Amphritea* (Fig. 7B). The only significant and important (*r* > 0.25) negative interactions were observed between *Roseibacillus* and Mf105b01 and between SM1A02 and *Profundimonas*.

**FIG 8:**
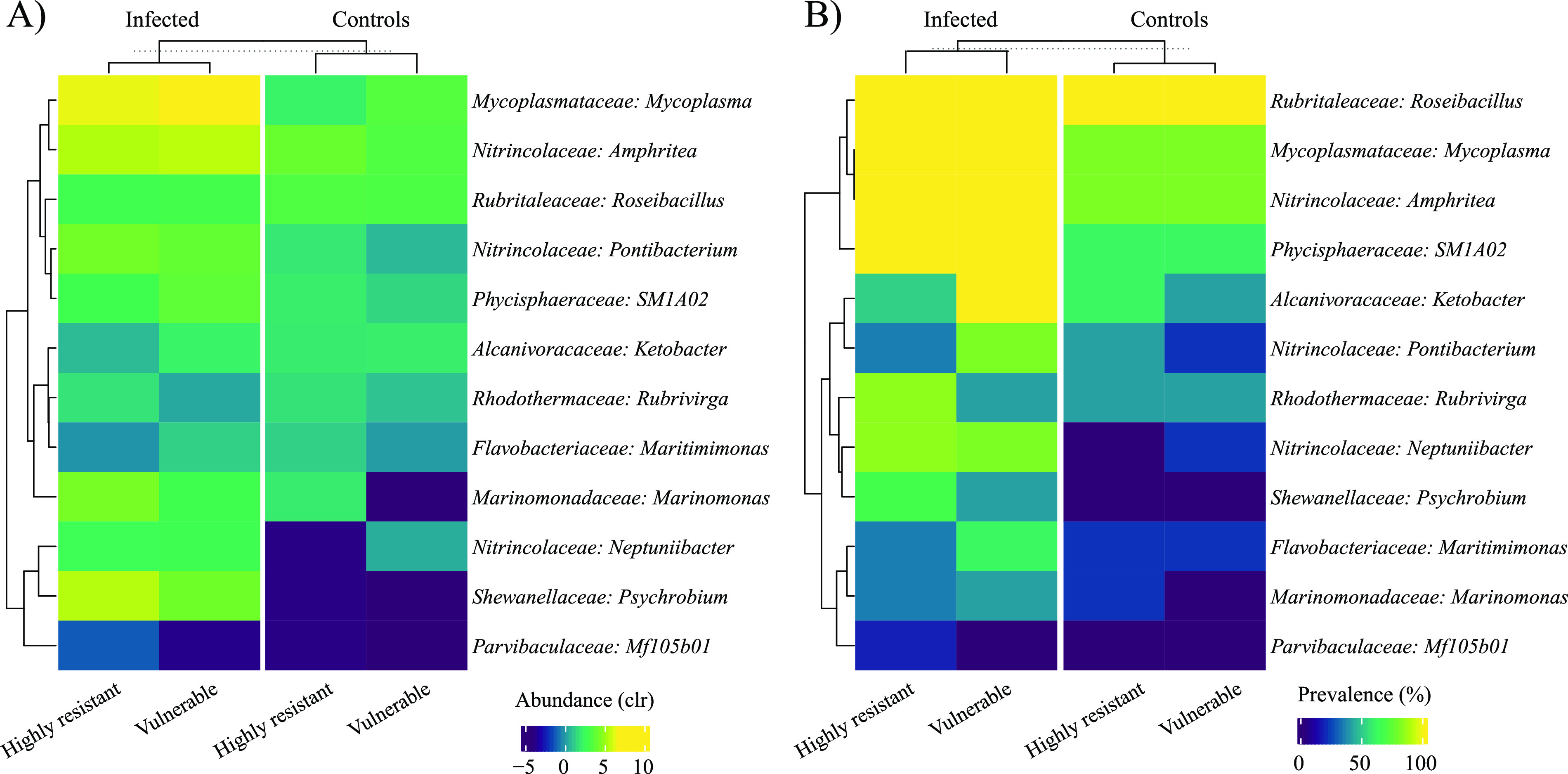
Microbiota relative abundance (A) and prevalence (B) between vulnerable and highly resistant families at genus level. Only genera with a prevalence above 30% and among the 30 most abundant are displayed.

Figure 8 shows the relative abundance (A) and the prevalence (B) of bacterial genera correlated with mortality rate, viral gene expression, or viral load between vulnerable and highly resistant families in control or infected conditions. Interestingly, infection induced an increase in the relative abundance of *Mycoplasma*, *Amphritea*, and *Psychrobium*, whereas the prevalence of *Mycoplasma*, *Amphritea*, SM1A02, and *Neptuniibacter* was increased in infected oysters. Noticeably, the prevalence of *Ketobacter*, *Pontibacterium*, and *Maritimimonas* strongly increased only in vulnerable families as opposed to the prevalence of *Rubrivirga*, which only increased in highly resistant families (Fig. 8B). Figure 8 also shows that most of the following taxa associated with POMS disease are present at low abundance in oysters before infection: *Mycoplasma*, *Amphritea*, *Ketobacter*, *Maritimimonas*, or S1A02.

**FIG 9:**
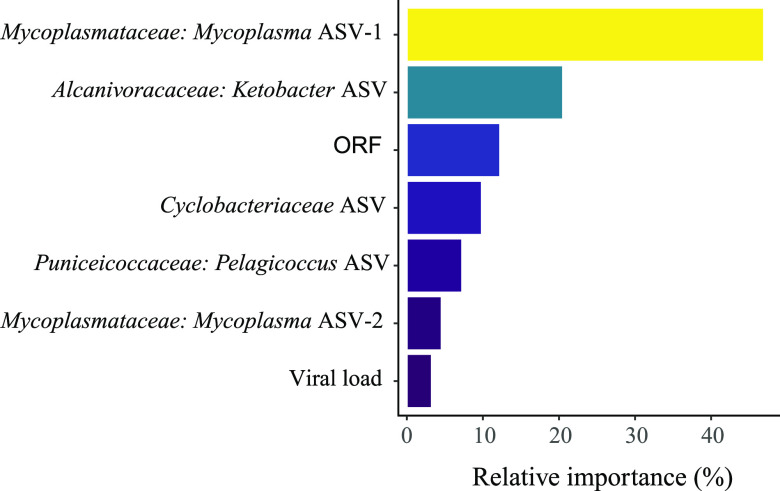
Relative importance of variables selected by a random forest analysis to predict mortality rate.

Figure 9 shows variables selected by random forest analysis to predict the outcome (positive or negative) of POMS disease. Presence of *Mycoplasma* ASV-1 and *Ketobacter* in oyster tissues were the best predictor of the POMS outcome (>60% relative importance), followed by ORF, *Cyclobacteriaceae*, *Pelagicoccus*, and a second *Mycoplasma* ASV-2. To a lesser extent (<5% relative importance), viral load in the surrounding water could also help predict mortality rate.

## DISCUSSION

In this study, we characterized the pathogenesis of POMS, the disease progression, and the associated changes in the microbiome of Pacific oysters from 10 contrasted families with variable genetically based resistance to POMS, following a lab-based infection with OsHV-1. Our main findings are (i) a delay in viral infection resulting in a late onset of mortality compared with previous descriptions and (ii) an absence of major fatal bacteremia in oysters during POMS. Further, comparison of microbiota carried by the different vulnerable and resistant families allowed (iii) the identification of potentially harmful and beneficial bacterial taxa that may influence the outcome of the disease.

### Temporal dynamics of POMS following OsHV-1 exposure in New Zealand.

In vulnerable families, mortality started 48 h after oysters were exposed to 10^9^ copies of OsHV-1 L^−1^, while significant mortalities (>10%) were recorded after 144 h to ultimately reach 50% at the end of the challenge (336 hpi). These results indicate a delayed viral infection in New Zealand compared to recent descriptions in Australia, the Unites States, and France where the thresholds of 10% mortality were recorded at, respectively, 48 hpi (36, 37), 96 hpi (38), and 120 hpi (13, 19) in studies using similar methods of infection and viral doses. In our study, delayed mortalities coincided with later expression of 3 common viral genes recorded after 96 h. In France, high expression of the same viral genes (ORF27, 38, and 87) was detected in challenged oysters earlier from 24 to 48 hpi (13, 19, 39). However, in our experiment, viral load was maintained around 6.5 × 10^8^ copies L^−1^ even after new filtered and UV-treated seawater was reintroduced in the recipient tanks at 48 hpi, suggesting that active viral gene expression and shedding of virions was occurring from 72 hpi.

Histological analyses performed after 72 hpi did not reveal any hemocytosis, diapedesis, inflammation, or bacterial colonization in tissues as previously described in oysters infected with OsHV-1 (13, 40, 41). Large, edematous areas resulting in the disorganization of the connective tissue in the mantle and digestive gland were observed, however, indicating a deterioration of the general health of infected animals. Additionally, hemocyte “blebbing” in the connective tissue of the digestive gland was observed more frequently in oysters exposed to OsHV-1. Such blebbing may be endocytosis of OsHV-1 particles (micropinocytosis [39–41]) or may be indicative of viral release by budding through the nuclear envelope (42). Alternatively, the hemocyte blebbing may be the early stages of apoptosis (zeiosis [43–46]); however, although nuclear fragments, which are indicative of apoptosis, were observed in hemocyte protrusions occasionally (Fig. 4D), the majority did not contain obvious nuclear fragments (indicated by deep blue staining; Fig. 4C, E, and F), and there was no notable increase in apoptosis or phagocytosis in this tissue.

### Changes in microbiota during POMS infection.

In the present study, we found distinct microbial taxonomic composition between oyster tissue and surrounding rearing seawater, a finding in agreement with other previous studies (33, 43, 44). Despite maintaining oysters under common and controlled conditions (i.e., seawater UV treatment, constant temperature, optimal dissolved oxygen, hatchery-cultured algal feed), we also found significant changes in the composition of oyster microbiota over time throughout the experiment, while bacterial composition and richness in the seawater microbiota were not affected by time, suggesting that oysters have a dynamic microbiome that can be selectively colonized by transient distinct bacterial taxa.

The oyster microbiota may also respond to or be modulated by stress experienced by the host, shifting toward an opportunist-dominated community that can affect the host’s fitness and survivability. This is exemplified by OsHV-1 infection in the Pacific oyster, which induces an immune suppression followed by the colonization of opportunistic bacteria in tissues, resulting in a dysbiosis that can lead to systemic infection and host death (13, 45). This phenomenon is typically characterized by the proliferation of one or very few bacterial species leading to a drop in alpha diversity and an increase in microbial dispersion (13, 46).

Surprisingly, in the current study, bacterial profiling analyses did not show marked changes of alpha diversity in oysters or water following OsHV-1 infection. However, significant changes in microbial beta diversity were induced by infection regardless of oyster vulnerability phenotype. During infection, microbial community composition of oyster tissues was strongly correlated with viral load, viral gene expression, and survival rate as shown by principal-coordinate analysis (PCoA)/Bray-Curtis statistical analyses. Furthermore, a limited number of bacterial families were significantly associated with mortality. Among these families, *Mycoplasma*, *Marinomonas*, *Psychrobium*, *Amphritea*, and *Neptuniibacter* were the most abundant. These specific taxa have been previously described as opportunistic pathogenic bacteria contributing to systemic infection during POMS (22, 23, 25, 47), supporting the conservation of the POMS pathobiota across geographically distant environments and varied oyster genetic pedigrees (22, 47).

Importantly, two specific *Mycoplasma* strains were found to be among the most important bacteria in predicting mortality following an OsHV-1 infection. *Mycoplasma* has the smallest known prokaryotic genome and, consequently, is believed to be an obligate commensal or parasite due to having limited metabolic capabilities (48). It has been found in high proportions in various oyster species across a broad geographic range and has been considered as a core microbe of the oyster’s gut tissue (25, 49–51). Nonetheless, some *Mycoplasma* strains are believed to become intracellular pathogens under environmental stress and to cause infections in bivalve molluscs (52–55). Interestingly, *Mycoplasma* has apical bleb-like protrusion that helps it conglomerate or attach to host cells (53), potentially explaining the “hemocyte blebbing” in the connective tissue of the digestive gland observed in infected oysters.

Furthermore, we found that bacteria from the *Profundimonas* genus were highly correlated with intense viral gene expression; presence of this taxa was only evidenced in Australia in the bacterial core of oysters presenting moderate mortalities following field exposure to OsHV-1 (23). Our study also characterized seven new bacterial genera in vulnerable oysters, all potentially implicated in the pathogenesis in New Zealand and not previously reported. Specifically, four members of the *Gammaproteobacteria* class, consisting of *Ketobacter*, *Pontibacterium*, *Profundimonas*, and *Nitrosococcaceae*, and two members of the phylum *Bacteroidetes* (*Algoriphagus* and *Maritimimonas*) were identified. Network association analyses highlighted complex patterns of interrelationships between these bacterial taxa and corresponding phenotypes like viral load, viral gene expression, and survival rate, suggesting a possible bacterial consortium associated with host colonization as previously described in France (47).

All together, these data reveal some variations in the course of the disease compared to previous descriptions carried out in other countries. Despite a high viral concentration in the water, high mortality rates recorded in vulnerable families, and the presence of *Mycoplasma*, *Amphritea*, *Pontibacterium*, *Ketobacter*, and *Maritimimonas* associated with infected individuals, viral gene expression (based on ORF27, 38, and 87) occurred later than in other comparable studies, and oysters did not experience massive dysbiosis. In addition, amplicon sequencing did not reveal a notable abundance of taxa from the genus *Vibrio* associated with mortality. This finding differs from previous studies where naturally infected oysters commonly showed an increase in the load of *Vibrio* spp. as the disease progresses (10, 26, 28, 46). The use of hatchery-born, OsHV-1-free oysters combined with the use of a purified isolate of OsHV-1 for donor injection and filtered, UV-sterilized seawater could explain the low prevalence of *Vibrio* spp. observed. Further research involving methodologies with higher taxonomic resolution (e.g., metagenomics) would be necessary to adequately identify the putative pathogenic strains detected in this study.

### Is there a distinct OsHV-1 variant specific to New Zealand?

We can hypothesize that the delayed timing in the course of infection, the limited dysbiosis, and the unusual features of hemocytes via histological observations may be the result of a distinct New Zealand OsHV-1 variant. During a mortality episode of Pacific oyster juveniles in the summer of 2010 to 2011, Keeling et al. (10) analyzed the C2-C6 region of the OSHV-1 virus isolated in the North Island of New Zealand. The authors reported that the isolated sequence shared similar variations with the reference OsHV-1 μVar. However, the New Zealand specimens were also carrying variations of two nucleotides common to the OsHV-1 reference strain (GenBank accession number AY509253) diverging from μVar (10). Viruses generally exhibit high levels of genetic diversity, having the ability to produce diverse and genetically linked mutants. The level of *de novo* genetic diversity within viral populations likely influences viral pathogenicity, host determination, dissemination, and host immune evasion (56). Prior to 2008 and the identification of OsHV-1 μVar in France (7), the following two genotypes of OsHV-1 were identified: the reference strain (57) and a second genotype OsHV-1 Var detected in *Pecten maximus* (58). With the recent advances in genomic sequencing efforts, considerable genotypic variations within the OsHV-1 species have been established. These variations are associated with different host species as well as temporal and geographical factors (59–61), supporting the possible emergence of specific variants in New Zealand. The current results indicate that genomic sequence comparison of strains between New Zealand, Australia, Europe, and the United States may indeed be a fruitful area for future research.

### Differences between vulnerable and resistant families.

Another explanation for the different dynamics of viral infection and the absence of bacteremia or apoptosis observed in oysters in the current study may be that what we term “vulnerable” families could still have inherited some resistance traits from their parents. Indeed, the females used to produce our vulnerable families were a subset of oysters from families that showed poor survival but still survived at least two on-farm challenges to OsHV-1. Indeed, host immune ability to defend against OsHV-1 has been found to have a genetic basis in the Pacific oyster with resistance shown to be moderately to highly heritable (14, 15, 62). Similarly, resistance against bacterial infection, such as Vibrio aestuarianus, has also been found to have a genetic component with moderate heritability (14), further highlighting the potential of selective breeding for increased disease resilience.

In this study, no significant difference in host-microbial taxonomic composition could be observed between family types (i.e., vulnerable, resistant, highly resistant). Nonetheless, *Neptuniibacter*, a bacterium strongly correlated with mortality rate and viral load, was solely found in vulnerable families, albeit in low abundance and prevalence. Interestingly, taxa highly associated with mortality rate and viral load, such as *Ketobacter*, *Mycoplasma*, and *Psychrobium* were similarly present in control specimens across family types (Fig. 7), indicating that their potential to cause a disease was reduced in highly resistant families. While no significant difference could be observed between family types, we did observe a significant effect of family (i.e., F1, F2, F4, etc.).

Moreover, among the 10 oyster families exposed to OsHV-1 under common and controlled conditions, we obtained a range of mortality dynamics, survivorship (ranging from 99.3% to 50.8%), and viral gene expression (magnitudes of ORF expression from 0 to 10,000 in relative expression). For example, family 1 exhibited an OsHV-1 resistance (i.e., ability to control pathogen burden, 99.3% survival, and very low viral gene expression), while families 2 and 3 seem resilient to the infection (i.e., ability to maintain performance while infected, medium viral gene expression, and survival of >82%). This illustrates the complexity of POMS in the Pacific oyster and, together with eventual specific variation of OsHV-1, may explain the diversity of host × pathogen interactions observed in different producing countries worldwide. Molecular analyses (transcriptome sequencing) of the mechanisms underlying the contrasted responses to the disease and genome-wide association studies (GWAS) would greatly contribute to the identification of valuable candidate genes for selective breeding and improve productivity in the presence of POMS.

To conclude, characterization of bacterial communities associated with oyster spat infected with POMS in a controlled environment can assist in understanding the role and function of the microbiome in disease resistance in C. gigas. The development of selective breeding in aquaculture (63) will also provide increasing opportunities to access material showing contrasted phenotypes, allowing for a better understanding of the molecular bases of complex traits, such as resistance to POMS in *C. gigas*. Collectively, these results could improve current disease management and aquaculture practices. Furthermore, the use of 16S rRNA gene sequencing/metabarcoding and the identification of bacterial species as a predictive factor to determine survival of oysters may open new perspectives as a phenotyping tool during selective breeding and/or a diagnostic tool to determine shellfish health.

As with any microbiome study, there are limitations in amplicon sequencing, and deriving conclusions on relative (nonabsolute) bacterial proportion and quantification should be done cautiously. Future studies should include targeted quantification of specific bacteria or attempts to normalize ASV abundance with quantitative PCR (qPCR) of the total bacterial community (24, 25). Metagenomic association-wide study could also help us identify the key genes from specific strains that may be negatively affecting oysters’ health, revealing essential clues on their role in pathogenicity and adaptability.

## MATERIALS AND METHODS

### Pacific oyster family production and maintenance.

Ten biparental Pacific oyster (Crassostrea gigas) families were produced in March 2019 at the Cawthron Institute’s hatchery in Nelson, New Zealand (41°11'33.3"S, 173°21'37.8"E). Broodstock selected for spawning originated from the Mahurangi harbor (Warkworth, New Zealand; 36°25'28.16"S, 174°41'32.36"E). Parents used to produce resilient families have been selected for three generations and were survivors from full-sib families that had been exposed to an on-farm virus challenge and selected based on their high survival rates in the field (15). Parents used to produce susceptible families were derived from (i) a subset of the families that showed poor survival during the on-farm challenge and (ii) specific males that were reared on an uninfected farm on the South Island, and were, therefore, expected to be naive and highly susceptible to the virus (15). Cryopreserved sperm from these individual naive males were thawed according to Adams et al. (64).

Twenty million eggs from each female were fertilized with either fresh sperm or thawed sperm from a single male. Resulting embryos from a single cross were then incubated separately for 24 h according to Vignier et al. (44). Individual families were reared using a high-density larval rearing flowthrough system in the hatchery for 5 weeks according to a modified protocol from Ragg et al. (65).

Oyster families were then further on-grown for 5 months in a common flowthrough water tank but kept separate in family-specific upwelling tanks. All families were reared under common conditions as follows: seawater temperature ranged from 9°C to 18°C, and algal food was continuously supplied to provide an optimal growing environment for the spat. Husbandry treatments, such as grading and biomass adjustments, were conducted simultaneously between families. Despite these measures, mean liveweight of spat at the beginning of the trial varied among families (see Table S6 in the supplemental material). In the nursery tank, oyster families were continuously supplied with UV-sterilized seawater (80 mJ cm^−2^) and maintained under strict biosecurity management to ensure that no pathogen would interfere with later experiments. The “pathogen free” status of the experimental oysters was confirmed prior to the initiation of the experiment; indeed, no OsHV-1 DNA was detected using qPCR (*n* = 100 [66]). Finally, no significant spat mortality was observed prior to the start of the experiment.

### Experimental design. (i) Acclimation of oysters.

On 4 September 2019, virus-free oysters were randomly collected from their family-specific upwelling tanks and transferred to the experimental challenge facility at the Cawthron Institute (Nelson, New Zealand; 41°16'16.7"S, 173°17'36.3"E) for acclimation.

**FIG 10:**
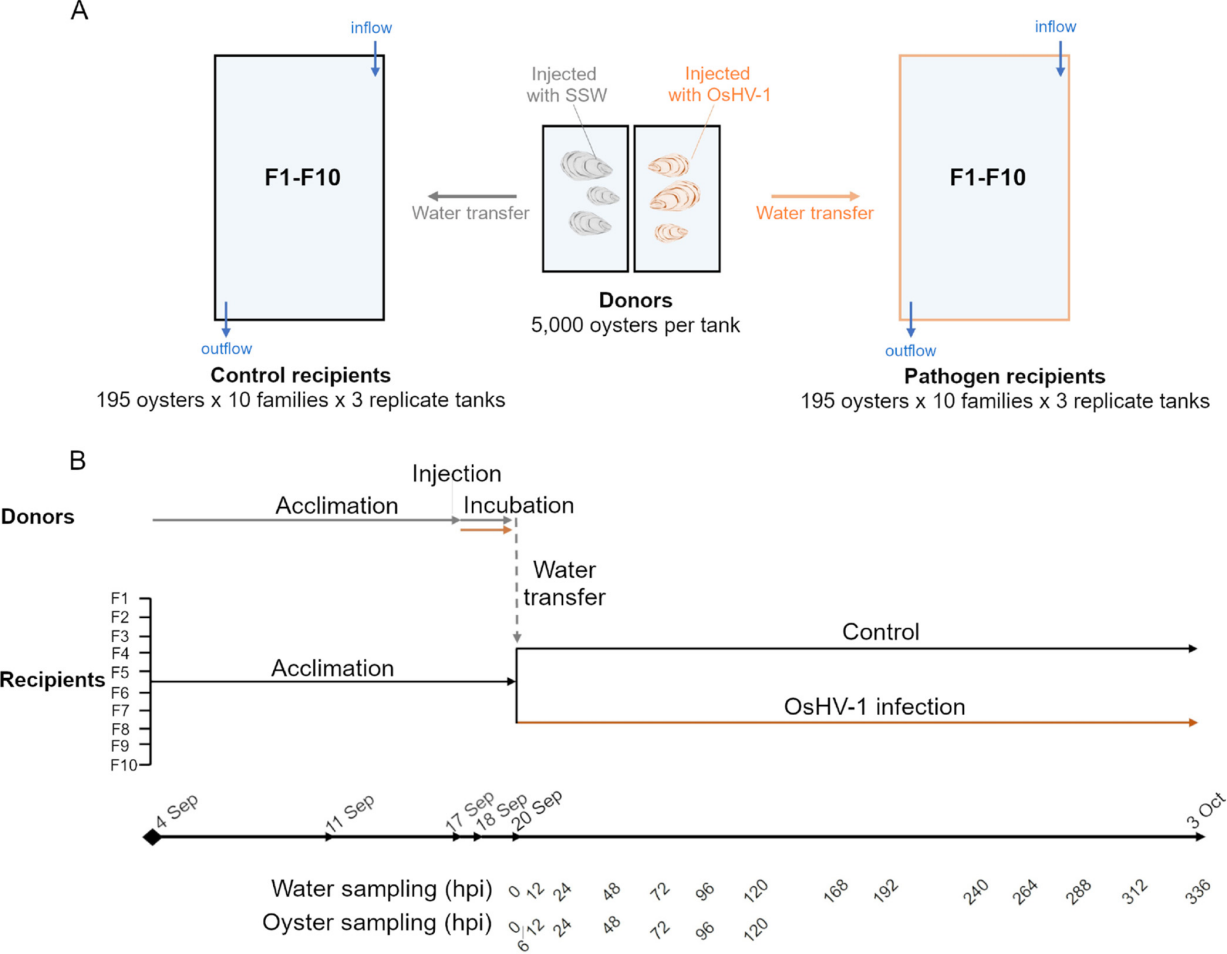
Experimental design to investigate the disease progression and the susceptibility of 10 oyster families exposed to ostreid herpesvirus type 1. (A) Donors and recipient oysters. (B) Experimental timeline.

Experimental infection protocols consisted of a water transfer between tanks containing *C. gigas* oysters carrying the disease (referred to as “donors”) and tanks containing pathogen-free oysters (referred to as “recipients”) (adapted from references 19, 21, 67). Two pools of 5,000 spat each (6-month-old oysters, mean individual weight of ~1.2 g), consisting of a mixture of oysters from our susceptible families, were used as donors and placed in two 300-L tanks, one tank for the control donors (which were to be injected with artificial seawater) and one tank for the pathogen donors (which were to be injected with a suspension of purified isolate of OsHV-1) (Fig. 10). Tanks were continuously supplied with 1-μm-filtered and UV-sterilized seawater (FSW), preheated through a Digiheat inline heater (Waterco, Auckland, New Zealand), and flow rates were maintained at 1.5 L min^−1^ per tank. Donors were maintained in these conditions for 2 weeks until their injection.

In parallel, family-specific mesh bags containing 195 oysters (6-month-old spat, mean individual weight of ~1.2 g) were prepared for each of the 10 families and used as recipients. One bag per family was randomly suspended (i.e., 10 families × 1 bag = 10 bags per tank) into six 100-L tanks. Three replicate recipient tanks were set up for the pathogen group and three replicates for the control group (Fig. 10A and B). Total biomass per recipient tank (corresponding to 1,950 spat) was 2.4 ± 0.2 kg. Flow rates were adjusted to 0.5 L min^−1^ for each tank by means of a valve, so combined flow rate for the triplicate tanks was 1.5 L min^−1^. Recipient oysters were acclimated under these conditions for 15 days until viral inoculation. No mortality was observed during this time.

To thoroughly maintain seawater temperature at 20.8°C (±0.8), oxygen saturation above 90%, and seawater well homogenized, all tanks were equipped with an aquarium immersion heater (Eheim thermocontrol 200W), light aeration, and a circulation pump (Hailea, low water level pump DS-700). All oysters (donors and recipients) were fed *ad libitum* with a bispecific mixture of hatchery-grown Chaetoceros muelleri (CS-176) and Tisochrysis lutea (CS-177) continuously supplied using a peristaltic pump. Algal background concentration was maintained between 3 and 10 μg L^−1^ Chlα (equivalent to 5 to 20 cells of *Tiso* per microliter).

### (ii) Viral suspension.

The OsHV-1 suspension stock was produced in April 2014 as described in Camara et al. (15) and adapted from Kirkland et al. (68). Briefly, whole tissue from high-virus-load oysters was homogenized, cell debris was removed by centrifugation, and the supernatant was purified by serial filtrations to 0.22 μm. Finally, a cryoprotectant solution (10% glycerol and 10% fetal calf serum final concentration) was added, and the resulting suspension slowly frozen and stored at −80°C.

### (iii) Infection and sampling procedures.

On 17 September 2019, pathogen-donor oysters were myorelaxed in hexahydrate MgCl_2_ (30 g L^−1^) according to reference (69) until valve opening. Concurrently, cryopreserved virus stock suspension was thawed in a 22°C water bath for 10 min and diluted 1/5 in sterile artificial seawater (SSW). Pathogen donors were then injected in the adductor muscle using a 26-gauge needle attached to a multidispensing hand pipette, with 20 μL of viral suspension (1.76 × 10^5^ copies of OsHV-1 per injection), while “control donors” were injected with the same volume of SSW. Oyster donors (pathogen and control) were then held at 22°C (±0.5) for 70 h in their respective tanks in static conditions (i.e., no water renewal) to produce infectious or control water. During the incubation period, survival of donor oysters was monitored daily, and dead animals were immediately removed from the tanks.

On 20 September, infectious or control water was transferred to the respective recipient tanks (3 tanks OsHV-1 challenged and 3 tanks control). Feeding was stopped, and the recipient tanks were left without flowthrough at 22°C (±0.5). After 48 h, new FSW enriched with microalgal feed (70) was gradually reintroduced to the 100-L recipient tanks at a flow rate of 0.5 L min^−1^. Each bag of recipients was emptied and thoroughly inspected daily, and any dead animals, characterized by failure to close their valves, were immediately removed. Survival of recipients was monitored for 14 days or 336 h postinfection (hpi). To avoid accidental release of OsHV-1 to the environment, continuous chlorination (200 ppm for 1 h) combined with UV sterilization (80 mJ cm^−2^) were applied to the effluent water.

Five live recipient oysters were randomly sampled from each family-specific bag at 0, 2, 6, 12, and 24 hpi and every 24 h until 120 hpi. A final sampling of 5 live recipient oysters was conducted at the end of the challenge, at 336 hpi (Fig. 10). Whole tissues were removed from the shells, dried by dabbing on a paper tissue, flash frozen in liquid nitrogen, and later reduced to powder (Mixer Mill MM400; Retsch GmbH, Germany) and stored at −80°C for OsHV-1 DNA, viral gene expression, and metabarcoding analyses. In addition, three live recipients were randomly collected from each family-specific bag at 0 and 72 hpi. Their tissues were carefully dissected, placed in histological cassettes, fixed in 4% formalin for 48 h and stored in 70% ethanol for later histopathological analysis.

One liter of seawater was collected from each donor tank (*n* = 2) at 0, 48, and 72 hpi, while 1 L of seawater from each recipient tank (*n* = 6) was sampled at 0, 24, 48, 72, 96, 120, 168, 240, and 288 hpi (Fig. 10). Water was collected using sterile (autoclaved) glass bottles and immediately filtered through a sterile 47-mm cellulose membrane filter of a 0.22-μm pore size to isolate bacteria and viruses. Membrane filters were flash frozen and stored at −80°C until DNA extraction.

### Biometric analyses and water quality measurements.

Each family-specific mesh bag was weighed on 4 September prior to acclimation (ST 1), on *T*_0_ (prior to viral on inoculation), and at the end of the challenge on 4 October (*T*_336h_).

Water quality parameters were measured throughout the challenge by means of a YSI ProSolo digital meter (Xylem Inc., Yellow Springs, OH, USA) for temperature and dissolved oxygen (DO), a handheld Testo 206 pH meter for pH, and a FluoroSense handheld fluorometer (Turner Designs, San Jose, CA, USA) for microalgal background levels.

### DNA extraction and OsHV-1 DNA quantification.

Total DNA was extracted from powdered donors (0 hpi), recipient oysters (0 and 120 hpi), and from seawater samples from donor tanks (0, 48, and 72 hpi) and recipient tanks (0, 24, 48, 72, 96, 120, 168, 240, 288, and 336 hpi) using a blood and tissue kit (Qiagen) according to the manufacturer’s protocol. Four blank DNA extractions were included in order to test for potential bacterial contamination of the DNA extraction kit and/or reagents.

Level of OsHV-1 DNA was quantified in water from both donor (0, 48, and 72 hpi) and recipient tanks (0, 24, 48, 72, 96, 120, 168, 240 hpi) using real-time PCR (Table 1). Real-time PCR was carried out in a 20-μL reaction mixture consisting of 10 μL of SsoFast EvaGreen Supermix (Bio-Rad), 1 μL of each primer (OsHV-1 BF and OsHV-1 B4, 10 μM), 6 μL of water, and 2 μL DNA sample. PCR amplification was performed using Rotor-Gene Q (Qiagen) following 1 cycle preincubation at 98°C for 2 min, 40 cycles of amplification at 98°C for 15 s, and 58°C for 20 s as well as melting temperature curve ramping from 72°C to 95°C, rising by 1 degree each 5 s. Samples were analyzed in triplicate, and 3 controls were carried out as follows: a negative control which contained PCR mixture without the target, an extraction control, and a positive control which holds DNA target(s). OsHV-1 DNA concentration in the sample was assessed using a quantitative curve for the standard (plasmid pOsHV1-Breg [71]).

### Microbiota sequencing.

Polymerase chain reactions (PCR) were performed on some of the samples extracted as described earlier (*n* = 120 from oyster tissue; *n* = 54 from water, plus blank controls). Bacterial communities were amplified using the 16S rRNA gene (v3-v4 region) with the primer set 341F, 5′-CCT ACG GGN GGC WGC AG-3′ (72), and 805R, 5′-GAC TAC HVG GGT ATC TAA TCC-3′ (73). These primers were modified to include Illumina overhang adaptors (forward, 5′-TCG TCG GCA GCG TCA GAT GTG TAT AAG AGA CAG-3′, and reverse, 5′- GTC TCG TGG GCT CGG AGA TGT GTA TAA GAG ACA G-3′) as described in Kozich et al. (74).

Polymerase chain reactions were carried out in 50-μL reaction volumes containing 25-μL MyFi 2× PCR supermix (Bioline, London, UK), 19 μL of nuclease-free H_2_O, 0.20 μM modified Illumina overhang adaptor primers, 2.0 μM both blocking primers, and 1 μL of template DNA. Thermocycling conditions were 95°C for 2 min, followed by 39 cycles of 94°C for 20 s, 52°C for 20 s, and 72°C for 30 s, with a final extension step at 72°C for 5 min. Amplicons were purified using the SequalPrep normalization plate kit (Applied Biosystems, CA, USA) resulting in an equimolar concentration of ~1 ng μL^−1^, all according to the manufacturer’s instructions. Purified amplicons were individually indexed using the Nextera DNA library prep kit (Illumina, CA, USA) and paired end sequenced on a MiSeq Illumina with the 2 × 250 bp v2 kit at Auckland Genomics (Auckland, New Zealand).

### Viral gene expression.

Viral gene expression was quantified in recipient oysters from the 10 families sampled at 6, 12, 24, 48, 96, and 120 hpi. RNA was extracted from 30 mg of powdered oysters using the Direct-Zol RNA miniprep kit (Zymo research) according to the manufacturer’s protocol. Samples were then treated with DNase I (Turbo DNase; Invitrogen) to remove genomic DNA. To confirm the absence of DNA in the sampled RNA, a 16S PCR assay was performed on each RNA sample after DNase treatment and gels were run. The quality and purity of the isolated RNA in all samples were checked using a NanoPhotometer (Implen, Munich, Germany). DNase-treated RNA was transcribed into cDNA, using the SuperScript III reverse transcriptase (Life Technologies, CA, USA). Droplet digital PCR (ddPCR) was conducted in an automated droplet generator (QX200 Droplet Digital PCR System; Bio-Rad) to determine the expression of three viral genes ORF 27, ORF 38, and ORF 87 selected from among the 39 ORFs described by Segarra et al. (39). These ORFs encode for different protein functions and expressed differently during an OsHV-1 replication cycle (39). Each ddPCR reaction included 1 μL of 3 μM each primer, 10 μL ddPCR Supermix for EvaGreen (Bio-Rad), 1 μL DNA, and 7 μL sterile water for a total reaction volume of 21 μL. The Bio-Rad QX200 droplet generator partitioned each reaction mixture into nanodroplets by combining 20 μL of the reaction mixture with 70 μL of Bio-Rad droplet oil. After processing, this resulted in a total nanodroplet volume of 40 μL, which was transferred to a PCR plate for amplification using the following cycling protocol: hold at 95°C for 5 s, 45 cycles of 95°C for 30 s, 60°C 1 s, and a final enzyme deactivation step at 98°C for 10 min. The plate was then analyzed on the QX200 instrument. For each ddPCR plate run, at least one negative control (RNA/DNA-free water; Life Technologies) and one positive control (Gblock for each ORF tested, diluted 1/10,000) were included.

### Histological analyses.

Recipient oysters were collected for histopathological analysis at 0 and 72 hpi. Three individuals per family (10 families at 0 and exposed oysters at 72 hpi and 3 families for nonexposed oysters at 72 hpi) were sampled per tank (3 tanks). Following storage in 70% ethanol, whole oysters were embedded in paraffin wax before being serially sectioned to a thickness of 5 μm using a microtome. Tissue sections were collected on polylysine-coated slides and stained with Giemsa (performed by Gribbles Veterinary pathology, New Zealand), which contains a mixture of azure and eosin that variably stain the basic components of the cell pink/purple (e.g., cytoplasm, granules) and methylene blue, which stains the acidic components of the cells blue (e.g., nucleus). The presence of histopathological features was assessed in the mantle and digestive gland of oysters using a light microscope and up to ×1,000 magnification (Olympus BX53 microscope with a DP22 digital camera). Pathological features were categorized as “0” when absent and “1” when present and were converted to a percent presence of a given feature per family, keeping tank replication (*n* = 3).

### Bioinformatic analysis.

Sequence data was demultiplexed using the MiSeq Reporter (version 2) and primers removed using Cutadapt (version 2.6) (75) allowing for no indels and a minimum overlap of 17 base pairs (bp). Sequences were quality filtered (maxN = 0, maxEE = c(2), trunQ = 2), denoised, paired end merged (minOverlap = 10), and chimera filtered (method = consensus) using the “DADA2” R program (version 1.14) (76). Prior to quality filtering, forward and reverse reads were truncated at 226 and 220 bp on the 5′ end, respectively, to remove the lower quality section. Amplicon sequence variants (ASVs) were taxonomically identified using the RDP Naïve Bayesian classifier algorithm (77) (implemented in DADA2 using the SILVA rRNA gene database (78)) (version 138; https://benjjneb.github.io/dada2/training.html). Unassigned ASVs and those identified as nonbacterial were discarded. Additionally, potential contaminant reads were identified and removed with the MicroDecon R package (version 1.0.2) (79), and rare ASVs (ASVs for which the sum of the read did not exceed more than 2 reads in at least 3 samples) were discarded. Sequencing depth per sample was visualized with the “rarecurve” function of the “vegan” R package (version 2.5.7) (80), and samples with less than 10,000 reads were discarded to ensure that samples used in downstream analyses had sufficient sequencing depth to recover most of the diversity.

### Statistical analyses.

Survival functions were computed according to Kaplan and Meier (1958) using RStudio 4.1.0 and the “Survival” R package (version 3.2-13) (81). Survival time was measured in hours from the injection for (i) donors (see Fig. S1 in the supplemental material) or (ii) from the onset of infection for the recipients (Fig. 10). The data were read as the number of dead oysters within each bag per tank at each count. Survival time curves were compared using the cox regression model (82) after adjusting for (i) injection (OsHV-1 or SSW) for donors or (ii) for family (F1 to F10), and infection level (OsHV-1 or control) for recipient oysters, considering the random effect of the tanks and bags. The survival of control recipient oysters was not included in the statistical model because it was 100%. The proportionality of hazards (PH) was checked based on Schoenfeld residuals (83).

Mixed-design, time-repeated ANOVAs were performed to assess differences in (i) OsHV-1 DNA load in water of recipient tanks according to family (10 levels) and time (8 levels) and (ii) percent presence of a pathological feature depending on family, time, and exposure to OsHV-1. The replication unit was the tank in which the 10 families were maintained. All mutual interactions among factors were tested, and Tukey’s honestly significant difference test was used as a *post hoc* test. The normality of residuals and homogeneity of variances were graphically checked. Statistical analyses were performed in R studio, version 4.1.0 (R; https://www.R-project.org/). For viral gene expression, heatmaps were constructed using Multiple Experiment Viewer software ([84] http://mev.tm4.org/#/datasets/upload).

Microbial taxonomic composition in seawater and in oysters was investigated and visualized at phylum and family levels using bar plots and the “ggplot2” R package (version 3.3.5) (85). Alpha diversity metrics such as ASV richness, Shannon, and Simpson indexes were computed with the “Phyloseq” R package (version 1.34.0) (86) and visualized with line plots using “ggplot2.” The effect of treatment, family type (vulnerable versus resistant versus highly resistant), collection date and their interactions on alpha diversity metrics of oyster microbiota was investigated with linear mixed-effects regressions (LMER) using the “lme4” R package (version 1.1.27) (87). Similarly, the effect of treatment and time after start of experiment and their interactions on alpha diversity metrics of seawater microbial communities were investigated with a LMER.

The effect of treatment on the oyster microbial community composition and structure was visualized with a principal-component analysis (PCA) and tested with a permutational analysis of variance (PERMANOVA) using the “vegan” R package (version 2.5.7) (80) with the following parameters: adonis2 (Bray-Curtis distance matrix of read abundance table transformed to relative abundance ~ infection × family type + time × family type, blocks = tank, permutations = 999, method = “bray,” by = “terms”). Differences in core oyster microbiome between vulnerable and highly resistant families were investigated with heatmaps using the plot core function of the “microbiome” R package (version 1.13.8) (88) and using the “ComplexHeatmap” R package (version 2.6.2) (89).

Oyster bacterial genera associated with oyster mortality rate, ORF, and viral load were investigated with Pearson correlation based on centered-log ratio transformed read abundance to account for the compositional nature of the data and visualized with a heatmap using the “ggcorrplot” R package (version 0.1.3) (90). In addition, interactions between these bacteria were assessed with the CoNet Cytoscape plugin (version 1.1.1) (91) using Pearson correlations and the Bonferroni multiple-test correction. Variables that could best predict mortality rate, including oyster bacteria ASVs transformed to centered-log ratio, viral load, and ORF concentration were identified using a Random Forest model trained [parameters, trControl = “repeatedcv,” number = 5, repeats = 3, search = “grid”; tunegrid = expand.grid(.try=c(1:8))] using the “caret” R package (version 6.0.88) (92) and visualized with bar plots using ggplot2.

### Ethics approval and consent to participate.

The study was conducted according to the guidelines of the Declaration of Helsinki and approval of the Animal Ethics Committee was not applicable for the use of oysters.

### Data availability.

Raw sequences and the data set(s) supporting the conclusions of this article are publicly available in the NCBI repository under accession number PRJNA832870.
