# Supplementary material for: Understanding the Dynamic of POMS Infection and the Role of Microbiota Composition in the Survival of Pacific Oysters, Crassostrea gigas
Source: Microbiol Spectr. 2022 Oct 31;10(6):e01959-22. doi: 10.1128/spectrum.01959-22 (PMC9769987; doi:10.1128/spectrum.01959-22)
Supplement: Supplemental file 1 — Fig. S1 to S3 and Tables S4 to S6. Download spectrum.01959-22-s0001.pdf, PDF file, 0.2 MB [file spectrum.01959-22-s0001.pdf]

Supplementary material

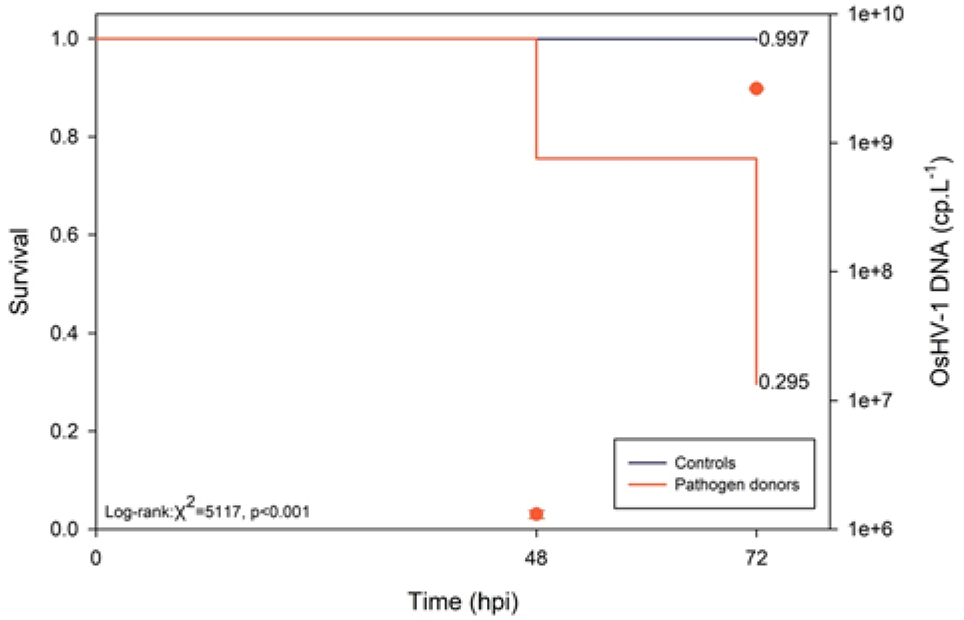

Sup Figure 1. Survival (solid line) of oysters injected with either OsHV-1 suspension (pathogen donors) or sterile seawater (controls) as a function of time (hours post infection; hpi). og-rank: Chi-square: 5117, p-value <0.001. The dots indicate the quantification of OsHV-1 DNA in the water, expressed as copies per litre. Data were log (x+1) transformed.

Sup Table 4: Permutational analysis of variance of oyster microbial community composition, with Infection, Family, Collection date, and their interaction terms as factors. Significant p values are highlighted in bold.

Term	R2	p value
Infection	0.097	**0.001**
Family	0.113	**0.001**
Collection date	0.036	**0.001**
Family * Infection	0.063	0.913
Collection date *Family	0.061	0.951
Residuals	0.629	

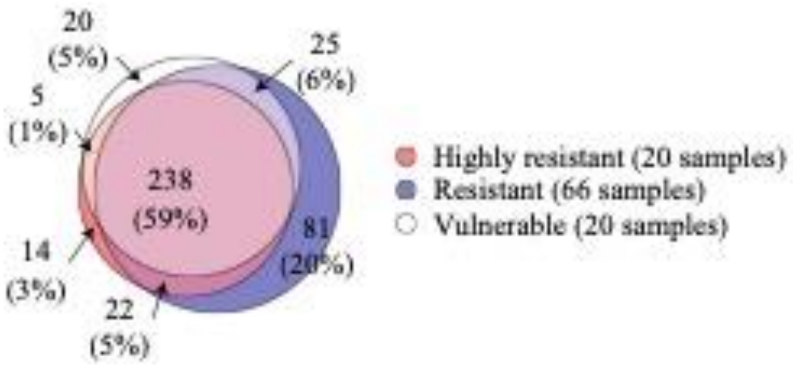

Sup Figure 2: Venn diagram of shared bacterial genera between oyster family types (Highly resistant, Resistant, Vulnerable).

Supplementary Table 5. Genera unique to each family type

**Vulnerable families**	**Highly resistant families**
Aerococcus	Arhodomonas
Alishewanella	Caminicella
Amaricoccus	Corallomonas
Arenibacter	MD3-55
Bosea	Nocardioides
Burkholderia-Caballeronia-Paraburkholderia	Paracoccus
Candidatus Amoebophilus	Pectobacterium
Clostridium sensu stricto 13	Pseudooceanicola
Delftia	Rhodococcus
Desulfosporosinus	Simiduia
Lactococcus	Sulfurimonas
Methylophaga	Sunxiuqinia
Nordella	Tistlia
Oceanisphaera	Waddlia
Pleurocapsa PCC-7319	
Psychrobacter	
Saccharospirillum	
Temperatibacter	
Terrabacter	
Treponema	

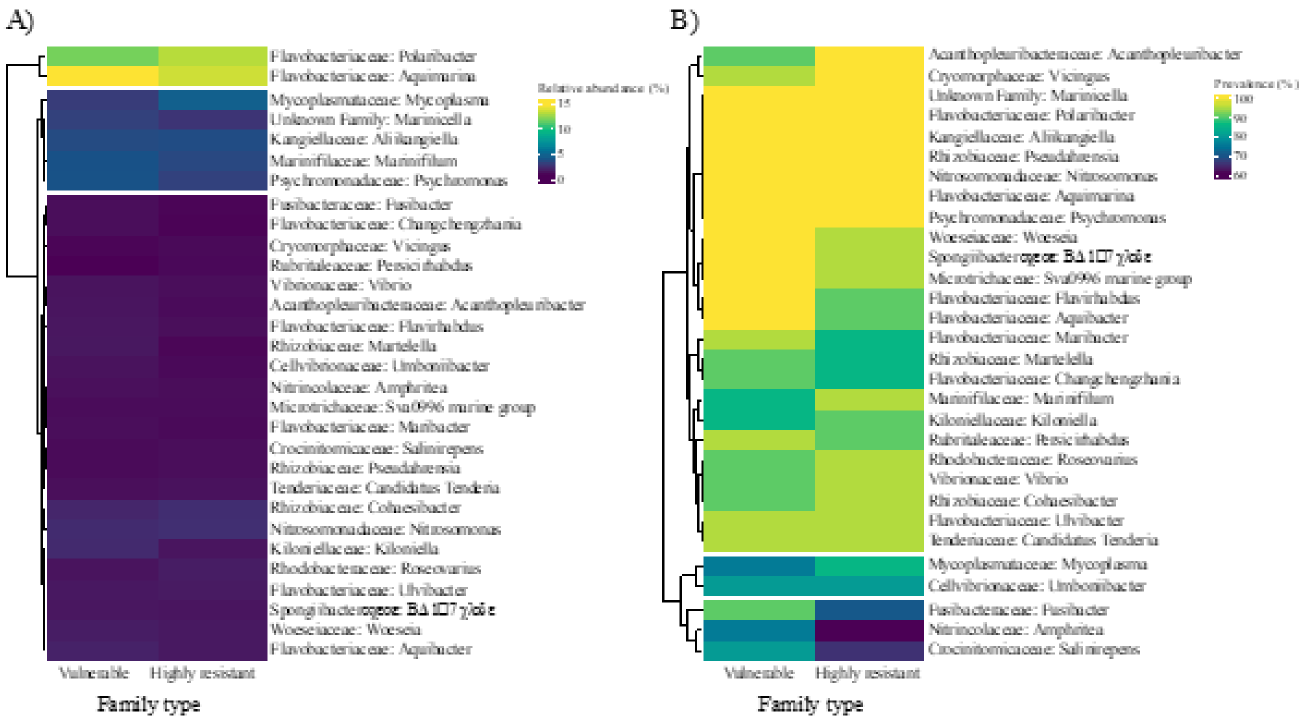

Sup Figure 3. Microbiome relative abundance (A) and prevalence (B) between vulnerable and highly resistant families at genus level. Only genera with a prevalence above 30 % and among the 30 most abundant are displayed.

Supplementary Table 6: Mean live weight (± SE), in grams, of ten families (F1 to F10) of recipient oysters from the control treatment, collected at the start (T0h) and the end of the experimental challenge (T336h).

Family	T0h	T 336h
F1	1.24±0.4	1.79±0.5
F2	1.03±0.3	1.2±0.2
F3	1.52±0.2	1.65±0.3
F4	1.06±0.3	1.36±0.5
F5	0.65±02	0.77±0.2
F6	1.11±0.3	1.69±0.9
F7	1.75±0.4	1.99±0.6
F8	1.34±0.4	1.6±0.9
F9	1.53±0.4	1.74±0.7
F10	1.41±0.3	1.60±0.6
